# Cerebral Blood Flow in Midlife Obesity: Associations with Visceral and Subcutaneous Abdominal Adipose Tissue

**DOI:** 10.1002/alz.093869

**Published:** 2025-01-09

**Authors:** Mahsa Dolatshahi, Paul K. Commean, Weiying Dai, Caitlyn Nguyen, LaKisha Lloyd, Sara Hosseinzadeh Kasani, Claude Sirlin, Bettina Mittendorfer, Tammie L.S. Benzinger, Joseph E. Ippolito, John C. Morris, Cyrus A. Raji

**Affiliations:** ^1^ Mallinckrodt Institute of Radiology, Washington University in St. Louis, St. Louis, MO USA; ^2^ Binghamton University, New York, NY USA; ^3^ Washington University in Saint Louis, Saint Louis, MO USA; ^4^ University of California, San Diego, La Jolla, CA USA; ^5^ Missouri University School of Medicine, Columbia, MO USA; ^6^ Washington University in St. Louis, St. Louis, MO USA; ^7^ Knight Alzheimer Disease Research Center, St. Louis, MO USA; ^8^ Washington University in St. Louis School of Medicine, St. Louis, MO USA

## Abstract

**Background:**

Obesity and higher adiposity in midlife are recognized as contributors to Alzheimer disease (AD). Neurodegeneration in AD is at least partly mediated by vascular compromise and brain hypoperfusion. In this study, we aimed to investigate the associations between BMI and abdominal visceral and subcutaneous adipose tissue (VAT, SAT) and brain cerebral blood flow (CBF) in cognitively normal midlife individuals.

**Method:**

A total of 66 middle‐aged cognitively normal adults (age: 49.86±5.99 years, females: 66.7%, obesity (BMI of 30 kg/m2 or higher): 51.5%, BMI: 31.72±6.96 kg/m2) underwent abdominal and brain MRI. Using an in‐house Matlab program, abdominal VAT and SAT were automatically segmented followed by manual editing. A 3D Pseudo‐Continuous Arterial Spin Labeling (pCASL) sequence, with a single post‐labeling delay of 2.025 s, was used for assessing CBF. SPM12 was used to generate ASL absolute CBF (aCBF) maps with a single compartment model, co‐registered to the gray matter segmentations, and normalized to MNI space, followed by spatial smoothing. Using AAL3 atlas and Matlab, region of interest masks were created for amygdala, hippocampus, posterior cingulate, precuneus, parahippocampal, medial orbitofrontal, and middle temporal cortices and applied to absolute CBF (aCBF) maps. The aCBF differences between the obese vs. non‐obese, high‐VAT vs. low‐VAT, and high‐SAT vs. low‐SAT was assessed, with age and sex as covariates. Also, BMI, VAT, and SAT as separate predictor variables, with age and sex as covariates, were used for voxel‐wise analysis.

**Result:**

There was a lower whole‐brain aCBF in the high‐VAT (p = 0.004) group and obese (p = 0.005) individuals, more prominently in the left middle temporal lobe (p = 0.002). No significant difference was observed in global and regional aCBF in the high‐SAT vs. low‐SAT groups. Voxel‐wise analyses showed significantly lower aCBF in association with BMI in temporal, occipital, and frontal lobe clusters after false discovery rate correction.

**Conclusion:**

Obesity and increased visceral abdominal fat are associated with a lower cerebral blood flow, with a more prominent decrease in the middle temporal cortex, as an AD‐signature area, in cognitively normal midlife individuals. These findings highlight the role of obesity, especially visceral obesity, in brain hypoperfusion and potentially Alzheimer disease risk, as early as midlife.

